# L'effet du COVID‐19 selon le sexe et le revenu: l'exemple des Philippines

**DOI:** 10.1111/ilrf.12226

**Published:** 2022-02-25

**Authors:** Rouselle F. LAVADO, Keiko NOWACKA, David A. RAITZER, Yana van der Meulen RODGERS, Joseph E. ZVEGLICH

**Affiliations:** ^1^ Organisation mondiale de la santé (OMS), (mais Banque asiatique de développement (BasD) à la date de rédaction de cet article); ^2^ BAsD; ^3^ Université Rutgers

**Keywords:** pandémie de COVID‐19, inégalités entre hommes et femmes, emploi, travail non rémunéré, pauvreté, confinement, femmes, Philippines

## Abstract

Les Philippines ont souffert plus que la plupart des autres pays en développement de la pandémie de COVID‐19 et des restrictions concomitantes, à savoir, notamment, un confinement très strict et une fermeture prolongée des écoles. Les auteurs proposent un modèle original pour estimer l'effet de ces mesures sur le travail, le revenu et la pauvreté selon le sexe et la région. Une étude de cas leur permet d’établir par ailleurs que les fermetures d’école ont nui à l'emploi dans l'enseignement privé et au revenu des parents de jeunes enfants. Ils concluent que la pandémie a eu des retombées économiques sans précédent et pénalisé les femmes.

## 1. Introduction: COVID-19 et genre

La pandémie de COVID-19 a d'énormes conséquences sur la santé et le bien-être des individus, des ménages et des sociétés du monde entier. De plus en plus de données témoignent d'une inégalité entre les sexes à cet égard: la pandémie fait plus de victimes parmi les hommes, mais d'autres dimensions de la maladie, non physiologiques, pénalisent davantage les femmes (Bahn, Cohen et Rodgers, 2020; Wenham, Smith et Morgan, 2020). Ainsi, la surreprésentation des femmes parmi les travailleurs essentiels, mal rémunérés et plus exposés au risque de contamination, est particulièrement préoccupante. Dans beaucoup de pays, les

* Organisation mondiale de la santé (OMS), lavador@who.int (mais Banque asiatique de développement (BasD) à la date de rédaction de cet article). ** BAsD; knowacka@adb.org, draitzer@adb.org et jezveglich@adb.org. *** Université Rutgers; yana.rodgers@rutgers.edu (autrice référente). Les auteurs tiennent à remercier Glenita Amoranto, Liming Chen et Xylee Javier pour leur précieuse contribution à la modélisation. Ils remercient aussi Esther Barrett et Yasuyuki Sawada, ainsi que les participants à l'édition 2021 du Forum des économistes de la BAsD pour leurs commentaires sur une précédente version de l'article. Les points de vue exprimés dans ce rapport sont ceux des auteurs et ne reflètent pas nécessairement les positions et les orientations de la BAsD ou de son conseil d'administration et des gouvernements qu'il représente.

Les articles paraissant dans la *Revue internationale du Travail* n'engagent que leurs auteurs, et leur publication ne signifie pas que le BIT souscrit aux opinions qui y sont exprimées.

Titre original: «COVID-19 disparities by gender and income: Evidence from the Philippines» (*International Labour Review*, vol. 161, nº 1). Traduit par Isabelle Croix. Révisé par la rédaction. Également disponible en espagnol (*Revista Internacional del Trabajo*, vol. 141, nº 1).

[Correction added on 26 March 2022, after first online publication: The copyright line was changed.]

femmes ont aussi été proportionnellement plus touchées que les hommes par les pertes d'emplois parce qu'elles sont très nombreuses à travailler dans les secteurs les plus durement frappés par la pandémie, comme le commerce de détail ou encore la restauration et l'hôtellerie (Alon *et al.*, 2020; BIT, 2021). À l'échelle mondiale, de plus en plus d'études révèlent que le travail de soin supplémentaire nécessaire en raison de la pandémie a principalement été assumé par les femmes (Craig et Churchill, 2021; İlkkaracan et Memiş, 2021; Zamarro et Prados, 2021). Par ailleurs, dans tous les pays, les travailleurs précaires et modestes ainsi que leur famille pâtissent davantage que les autres des mesures de confinement (Kabeer, Razavi et Rodgers, 2021). Les travailleurs migrants sont particulièrement vulnérables dans divers contextes, parce que leur situation, déjà précaire, est encore fragilisée par les interdictions de déplacement, l'exclusion des services publics d'urgence, les problèmes de logement, la violation de leurs droits en tant que travailleurs et les restrictions à l'immigration (Guadagno, 2020; Koh, 2020; Stevano *et al.*, 2021).

Les Philippines figurent parmi les pays en développement les plus durement frappés. D'après les données mondiales, le nombre des contaminations confirmées et des décès par habitant y était plus élevé que dans beaucoup d'autres pays d'Asie, et celui des cas diagnostiqués y atteignait un niveau record à l'échelle de l'Asie du Sud-Est. Au plus fort de la pandémie, en avril 2021, on y enregistrait ainsi 1 351 cas confirmés toutes les deux semaines, un chiffre supérieur à la moyenne mondiale (1 215 cas) et le plus élevé d'Asie après l'Inde[1]. Comme dans la plupart des pays, les taux de morbidité et de mortalité liés au COVID-19 sont plus élevés parmi les hommes que parmi les femmes: en avril 2021, 53 pour cent des cas confirmés et 60 pour cent des décès concernaient des hommes, à comparer avec des moyennes mondiales de respectivement 54 et 59 pour cent[2]. Toutefois, des données récentes laissent penser que la situation s'inverse en ce qui concerne les conséquences des restrictions imposées pour contenir la propagation du virus, à savoir que les femmes seraient plus touchées par les pertes d'emplois, assumeraient une plus grande part du travail non rémunéré supplémentaire et seraient plus souvent victimes de problèmes de santé mentale et de violences domestiques que les hommes (ONU Femmes et Women Count, 2020; Cho *et al.*, 2021; De los Santos et Labrague, 2021).

Comme dans beaucoup d'autres pays d'Asie, aux Philippines les pouvoirs publics ont décrété un confinement mi-mars 2020, et celui-ci a été l'un des plus stricts et des plus longs du monde (Hapal, 2021). La fermeture des écoles, notamment, devait durer jusqu'à ce que l'on ait pu vacciner toute la population (ce qui pourrait prendre plusieurs années), alors que partout ailleurs ou presque cette mesure avait été levée fin 2020 au plus tard (Raitzer *et al.*, 2020). Mais si les Philippines sont un cas d'étude intéressant, ce n'est pas seulement parce

[1] Hannah Ritchie, Edouard Mathieu, Lucas Rodés-Guirao, Cameron Appel, Charlie Giattino, Esteban Ortiz-Ospina, Joe Hasell *et al.*, base de données en ligne «Coronavirus Pandemic (COVID-19)», https://ourworldindata.org/coronavirus (consulté en avril 2021).

[2] Global Health 50/50, «The COVID-19 Sex-Disaggregated Data Tracker», base de données The Sex, Gender and COVID-19 Project https://globalhealth5050.org/the-sex-gender-and-covid-19-project/ (consulté en avril 2021).

qu’elles figurent parmi les pays en développement les plus durement touchés et que le confinement y a été particulièrement strict et prolongé. C’est aussi, en effet, parce qu’elles passent pour la nation la plus avancée de la région sur le plan de l’égalité entre hommes et femmes. Le pays est en effet doté d’une législation ambitieuse dans ce domaine et se classe dans le peloton de tête à l’aune de plusieurs indicateurs d’égalité importants, comme les salaires, l’éducation et la participation politique.

Dans cet article, nous examinons l’effet différencié du COVID-19 en fonction du sexe et du niveau de revenu dans le cas des Philippines. Nous utilisons à cette fin un nouveau modèle de simulation, reposant sur l’enquête nationale sur la population active et l’enquête nationale sur les dépenses et les revenus des ménages, afin d’estimer l’impact de la pandémie et des mesures de confinement sur le travail, le revenu et la pauvreté et d’étudier dans quelle mesure cet impact diffère selon le sexe, la région et le revenu. À travers notre étude de cas, nous cherchons également à analyser si les fermetures d’écoles ont eu des retombées supplémentaires sur le marché du travail, en termes de pertes d’emploi dans l’enseignement privé et de pertes de revenu pour les parents qui ont dû assurer la garde de leurs enfants et la scolarité à domicile. Nous constatons que la crise du COVID-19 et les mesures de confinement prises pour empêcher la propagation du virus ont eu des répercussions sans précédent sur l’activité économique des Philippines et ont davantage pénalisé les femmes que les hommes. Notre étude devrait aider les pouvoirs publics à prendre des décisions éclairées, de nature à favoriser l’égalité entre hommes et femmes et à faire reculer la pauvreté.

La suite de cet article est organisée comme suit. Dans la deuxième partie, nous brossons un tableau de la situation des Philippines. Dans la troisième, nous présentons le modèle situationnel utilisé pour estimer les conséquences de la pandémie et des mesures prises pour l’endiguer sur différents groupes, avant de décrire nos résultats dans la quatrième partie. La cinquième partie porte spécifiquement sur les retombées des fermetures d’écoles sur l’emploi et le revenu. Dans la sixième partie, qui fait office de conclusion, nous présentons une synthèse de nos constatations et des enseignements qui s’en dégagent pour l’action publique.

## 2. Contexte national

Les Philippines sont souvent présentées comme l’un des pays asiatiques les mieux classés selon plusieurs des principaux indicateurs d’égalité entre hommes et femmes, notamment le salaire, le niveau d’études et la participation politique. À titre d’exemple, en 2019, le pays occupait la 14$^{e}$ place sur 153 dans le classement du Forum économique mondial pour l’indicateur «participation et perspectives économiques» et le 29$^{e}$ rang pour l’indicateur de participation politique, devançant des pays voisins plus riches tels que le Japon, la République de Corée et Singapour (Forum économique mondial, 2019). Signe d’une participation relativement forte des femmes à la vie politique, 28 pour cent des parlementaires sont des femmes, contre une moyenne mondiale de 25 pour cent et une moyenne régionale de 20 pour cent (PNUD, 2020). Or, en 2021, le pays a perdu des places pour ces deux indicateurs du Forum économique mondial, occupant désormais les 18e et 33e places, ce qui pourrait s'expliquer par la sévérité du confinement imposé (Forum économique mondial, 2021). Par ailleurs, les Philippines ont réussi à atteindre une égalité entre hommes et femmes en ce qui concerne le niveau d'études et font partie des rares pays à faible revenu et à revenu intermédiaire dans lesquels la plupart des générations de femmes ont plus de chances que les hommes de suivre un enseignement formel. Comparativement à ce qui est observé dans d'autres pays ayant un niveau de développement comparable, les femmes qui occupent un emploi salarié sont relativement nombreuses à avoir suivi des études universitaires. Enfin, le pays est parvenu à une quasi-égalité de rémunération entre hommes et femmes, ce qui est rare dans le monde, et pas seulement parmi les pays appartenant au même groupe de revenu (BAsD, 2015; Zveglich, Rodgers et Laviña, 2019).

De l'avis général, la grande charte des femmes de 2008 constitue un instrument juridique majeur, instituant une obligation de lutte contre la discrimination, de protection des droits fondamentaux des femmes et de promotion de l'égalité entre hommes et femmes[3]. Ces avancées remarquables sont le fruit de politiques volontaristes et de l'action militante de la société civile sur fond de forte croissance économique. Or, malgré cette mobilisation et les résultats obtenus sur le plan de la rémunération, de la politique et de l'éducation, les Philippines sont en retard pour d'autres indicateurs, ce qui explique qu'elles n'arrivent qu'en 104e position dans le classement reposant sur l'indice d'inégalité de genre élaboré par le Programme des Nations Unies pour le développement (PNUD, 2020). Dans ce classement, elles font même moins bien que d'autres pays pour ce qui est de la santé procréative, comme en témoigne le niveau relativement élevé du taux de mortalité maternelle et du taux de natalité parmi les adolescentes. Le pays affiche aussi un taux d'activité féminine relativement bas par rapport à ses voisins de la région et à la moyenne mondiale *(ibid.)*.

La pandémie de COVID-19 représente donc un important test de résistance des progrès accomplis dans l'élimination des écarts entre hommes et femmes en ce qui concerne différents indicateurs d'autonomie, offrant notamment l'occasion de mesurer la capacité de ces avancées à survivre à un choc social et économique de grande ampleur. Elle constitue aussi l'occasion d'examiner si le pays a accentué son retard dans d'autres dimensions, comme le taux d'activité et la santé procréative.

À l'échelle mondiale, il est déjà admis que la pandémie menace les progrès accomplis sur le terrain de l'égalité entre hommes et femmes et pourrait anéantir des décennies de progrès. Car, si le taux de morbidité lié au COVID-19 est plus faible chez les femmes, celles-ci, ainsi que les filles, sont davantage pénalisées par les mesures prises pour empêcher la propagation du virus. Ainsi, selon les estimations du Bureau international du Travail (BIT), elles seront plus touchées que les hommes par les pertes d'emplois parce que 527 millions de femmes (soit 41 pour cent de l'emploi féminin total) exercent leur activité dans les secteurs les plus exposés, lesquels ne représentent que 35 pour cent de l'emploi masculin

3 Loi no 9710, Journal officiel, 14 août 2009.

(BIT, 2020)[4]. Par ailleurs, selon les prévisions, les fermetures d'écoles vont entraîner la non-scolarisation de 11 millions de filles (UNESCO, 2020). La fermeture des structures de garde d'enfants et des écoles a obligé les femmes à consacrer plus de temps aux activités non rémunérées, si bien que la répartition de ces tâches entre les membres du ménage est devenue encore plus inégale. En Asie et dans le Pacifique, les femmes leur consacrent en moyenne quatre fois plus de temps que les hommes (BIT, 2019). La perturbation des services de santé maternelle et infantile devrait se traduire par une hausse des taux de mortalité maternelle et infantile. Selon certaines estimations, le nombre de décès maternels devrait augmenter de 56 700 dans 118 pays à faible revenu et à revenu intermédiaire (Roberton *et al.*, 2020). De même, la violence dans le couple a augmenté sous l'effet des restrictions imposées et des difficultés sociales et économiques. Dans la région Asie et Pacifique, d'après les médias locaux et les organisations de la société civile, le nombre d'appels reçus par les services téléphoniques gratuits d'aide aux victimes de violence a connu une hausse comprise entre 33 et plus de 100 pour cent, et le nombre de femmes tentant d'accéder à des centres d'hébergement de victimes de violences domestiques a augmenté par rapport aux chiffres antérieurs à la pandémie (CESAP, 2020).

Les statistiques connues laissent penser que les Philippines n'échappent pas à ces tendances mondiales. Le groupe de travail interinstitutionnel pour la gestion des maladies infectieuses émergentes a pris des mesures de confinement sous la forme de quarantaines différenciées, prévoyant des restrictions différentes selon les secteurs d'activité en ce qui concerne les salariés autorisés à se rendre sur leur lieu de travail. La sévérité des mesures diffère également en fonction de la zone géographique concernée, des restrictions plus dures étant imposées dans les grandes métropoles, où les contaminations étaient plus nombreuses. La quarantaine la plus sévère (dite «quarantaine renforcée») s'apparente à un confinement. Lorsqu'elle est appliquée, la population ne peut quitter son domicile que pour faire des achats essentiels (nourriture et médicaments par exemple) et les salariés ne peuvent se rendre sur leur lieu de travail que si leur activité fait partie des activités essentielles. La «quarantaine générale» introduite en mai 2020 est moins stricte: elle permet de prendre les transports en commun pour autant que des jauges soient respectées et autorise les entreprises à fonctionner à condition que le nombre de travailleurs et de clients présents dans les locaux soit inférieur à la normale. La «quarantaine générale modifiée» a encore allégé les obligations et permet à la quasi-totalité des entreprises de fonctionner dans les zones où les contaminations sont peu nombreuses.

D'après les statistiques globales issues de l'enquête nationale sur la population active, le chômage est passé de 5,2 pour cent en avril 2019 à 17,6 pour cent en avril 2020, période à laquelle les fermetures d'entreprises se sont multipliées, et les hommes représentaient une plus forte proportion des chômeurs que les femmes. Au cours de la même période, le taux d'activité est passé de 61,3 à 55,7 pour cent, et les femmes représentaient la majorité des inactifs (PSA, 2020).

[4] Ces secteurs sont notamment les services d'hébergement et de restauration, les activités immobilières, commerciales et administratives, les activités de fabrication et le commerce de gros et de détail.

De plus, les données d’enquête indiquent que la pandémie a eu plus de retombées négatives sur la santé mentale des femmes, qui ont davantage souffert de stress, d’anxiété et de dépression (Tee *et al.*, 2020). La pandémie a aussi été source de problèmes et d’inégalités au sein du foyer. Les violences domestiques, qui constituent un problème ancien aux Philippines, se sont elles aussi aggravées sous l’effet des mesures de confinement strict (Sediri *et al.*, 2020). En outre, la quantité de tâches domestiques a augmenté et les femmes ont assumé la majeure partie de ce surcroît de travail, qui a essentiellement consisté à s’occuper des enfants et à les aider à effectuer leur travail scolaire, en particulier dans les zones reculées où la connectivité est faible et où les interactions avec les enseignants étaient inexistantes (Cho *et al.*, 2021). Une enquête réalisée auprès des ménages philippins montre que 40 pour cent des femmes consacrent davantage de temps à au moins trois tâches relevant du travail domestique non rémunéré en raison de la pandémie, contre 32 pour cent des hommes (ONU Femmes et Women Count, 2020). La même enquête révèle qu’aux Philippines les femmes ont, plus que les hommes, vu diminuer le revenu issu d’autres sources, notamment des envois de fonds des émigrés, de l’épargne et des entreprises familiales. Les pertes d’emplois ayant touché la diaspora philippine, majoritairement composée de femmes, l’économie du pays a enregistré une diminution de 14 à 20 pour cent des fonds envoyés de l’étranger par les travailleurs émigrés (Murakami, Shimizutani et Yamada, 2021).

La pandémie révèle au grand jour certaines des failles de la situation antérieure en matière d’égalité entre hommes et femmes, ce qui jouera un rôle déterminant dans le redressement à moyen et long terme. Les normes sociales relatives au rôle procréateur des femmes empêchent celles-ci de s’engager activement sur le marché du travail et contribuent à expliquer que le pays ait affiché, avant la pandémie, le taux d’activité féminine le plus faible de la région Asie-Pacifique. Selon une analyse réalisée pour le compte de l’autorité nationale chargée des questions économiques et de développement» (NEDA, pour National Economic and Development Authority), les Philippines sont le pays de l’ASEAN qui affiche le taux d’activité féminine le plus bas (46 pour cent). À cela s’ajoute que les femmes mariées ont une probabilité de travailler inférieure de 12 points de pourcentage à celle des femmes non mariées et que cet écart est encore plus grand pour les mères de jeunes enfants (Cabegin et Gaddi, 2019). Une vision traditionnelle de la répartition des rôles entre hommes et femmes et des normes sociales selon lesquelles il appartient aux femmes de s’occuper des enfants et aux hommes d’apporter le revenu du ménage conduisent davantage de femmes à quitter le marché du travail après leur mariage ou après la naissance de leur premier enfant.

D’autres dimensions de la santé sexuelle et procréative des femmes risquent d’accentuer les inégalités entre hommes et femmes. Avant la pandémie, les Philippines affichaient déjà l’un des taux de grossesse parmi les adolescentes les plus élevés d’Asie (5 pour cent), et 50 pour cent des femmes célibataires en âge de procréer indiquaient avoir besoin de services de planification familiale mais ne pas y avoir accès (PSA et ICF, 2018). D’après les estimations, on dénombrait en outre 751 000 autres grossesses non désirées en 2020 en raison des difficultés d’accès aux services de santé sexuelle et procréative (Marquez *et al.*, 2020).

L’augmentation du taux de natalité observée aux Philippines pendant la pandémie va encore accroître la charge qui pèse sur les femmes (Johnson, Simonette et Drury, 2020). Il est permis de penser que la pandémie de COVID-19 et les difficultés économiques qui en résultent vont accentuer ces phénomènes étroitement liés – tendances sur le marché du travail, normes sociales et tendances en matière de santé sexuelle et procréative –, avec les conséquences durables qui en découleront pour l’autonomie économique des femmes et l’égalité entre hommes et femmes.

## 3. Modèle de simulation

Dans notre analyse, nous recherchons d’éventuels effets sexués de la pandémie et des restrictions en modélisant les chocs de demande et d’offre associés à ces événements et les pertes d’emplois et variations des revenus concomitantes chez les femmes et les hommes. Notre modèle tient compte à la fois des pertes dues aux restrictions et de celles qui se seraient produites même en leur absence en raison de la peur du virus[5]. Suivant del Rio-Chanona *et al.* (2020), nous estimons les effets de chocs de demande et d’offre de premier ordre qui reflètent l’impact direct et indirect des restrictions imposées et des changements de comportement induits par le souci de ne pas s’exposer au virus. Nous n’estimons pas les effets globaux qui incluraient les conséquences secondaires des réductions ultérieures de la demande et de l’offre ainsi que des difficultés des chaînes d’approvisionnement mondiales[6].

Nous commençons par modéliser l’effet des chocs de demande et d’offre sur l’emploi. Ensuite, nous examinons comment ces chocs se traduisent en une diminution de la valeur ajoutée, puis du revenu, et nous utilisons ces variations pour simuler l’effet de la pandémie et des confinements sur la pauvreté.

### 3.1. Chocs de demande

Le COVID-19 exerce une forte influence sur les dépenses de consommation, même en l’absence de restrictions visant l’activité des entreprises. Outre que la demande de services de santé augmente, les consommateurs risquent de tenter de moins s’exposer au virus et de moins consommer de produits et services supposant des contacts sociaux étroits. Pour inclure ce comportement dans notre modèle, nous suivons l’approche de del Rio-Chanona *et al.* (2020) et faisons appel à des estimations établies par le bureau du budget du Congrès des États-Unis (CBO pour Congressional Budget Office) relativement à l’impact potentiel d’une pandémie de grippe (États-Unis, CBO, 2006). La pandémie modélisée par le CBO repose sur le syndrome respiratoire aigu sévère (SARS-CoV-1), et le modèle reflète les réactions comportementales qu’aurait la population pour éviter une contamination dans chaque secteur d’activité. Dans son analyse, le CBO part du

[5] Nous ne tenons pas compte des effets de la politique budgétaire, par exemple des aides versées au début de la pandémie dans le cadre du programme pour le progrès social (Social Amelioration Program).

[6] Pour le faire, il faudrait avoir recours à un modèle d’équilibre général, ce qui permettrait aussi d’inclure des scénarios tels que le retour au pays de travailleurs émigrés philippins.

principe que la demande de biens et services impliquant des risques de transmission – spectacles et voyages par exemple – diminue parce que les consommateurs cherchent à se protéger d'une éventuelle contamination. Il propose deux scénarios, l'un correspondant à un choc sévère et l'autre à un choc léger. Les chocs de demande sont prédits par secteur d'activité à partir de l'avis d'experts. Nous utilisons ces estimations parce que nous considérons que, même si la structure sectorielle de l'économie diffère aux Philippines et aux États-Unis, la population modifie vraisemblablement de la même manière sa consommation de certains biens et services en réaction au choc. Pour effectuer notre analyse, nous reprenons les estimations du CBO utilisées par del Rio-Chanona *et al.* (2020) et établissons une correspondance avec la nomenclature philippine des secteurs d'activité (PSIC pour Philippine Standard Industrial Classification).

Nous postulons que, dans un premier temps, les consommateurs réagissent à l'apparition du COVID-19 comme prévu par le scénario correspondant à un choc sévère. Le choc passe de sévère à léger entre mi-mars 2020 et la fin de l'année 2020, à mesure que la population s'habitue à la présence du virus et que la vague reflue.

## 3.2. Chocs d'offre

La diminution de l'offre de main-d'œuvre est plus ou moins grande selon les secteurs, les régions et les périodes. Elle est due aux mesures de distanciation sociale et d'interruption de l'activité imposées dans les secteurs à risque pour contenir la propagation du virus. Les obligations de distanciation sociale sont imposées pendant les périodes de quarantaine et dépendent de la proportion de travailleurs autorisés à se rendre sur leur lieu de travail à une date donnée d'après les annonces du groupe de travail interinstitutionnel. Les travailleurs qui restent chez eux ne sont pas nécessairement non productifs s'ils peuvent travailler à distance; cette possibilité dépend toutefois de leurs conditions de logement (il faut par exemple qu'ils disposent d'un espace de travail convenable et qu'ils aient accès à un ordinateur) et suppose que leur employeur accepte de recourir au télétravail et puisse le faire. C'est pourquoi nous supposons que, outre les travailleurs autorisés à se rendre dans leur entreprise, 50 pour cent de ceux qui exercent un métier compatible avec le télétravail travaillent effectivement depuis leur domicile. Les réductions relatives modélisées dépendent du degré de quarantaine appliqué dans chaque région et au cours de chaque période. Pour chaque région, nous retenons pour les différents secteurs des réductions de l'offre de main-d'œuvre qui dépendent des mesures de quarantaine imposées pendant la proportion de l'année 2020 à laquelle correspond la période étudiée. Ces mesures de quarantaine ayant principalement pour effet de réduire le nombre de travailleurs autorisés à être présents sur leur lieu de travail, elles sont modélisées sous la forme de chocs sur l'offre de main-d'œuvre.

Les Philippines étant un pays en développement, une grande partie de la production économique provient d'activités indépendantes ou du secteur informel. Pour inclure dans le modèle les effets qui en découlent, il faut modéliser les conséquences sur la valeur ajoutée $Y$ de manière à inclure la baisse de la demande de main-d'œuvre correspondante $L$. Les effets des chocs d'offre sont modélisés au moyen d'une fonction de production, dans laquelle nous considérons que le choc est un choc touchant le facteur travail. Nous considérons que la production proportionnelle d'un secteur $i$ dans la région $j$ au cours d'une période postérieure au choc correspond à la part de main-d'œuvre restante, élevée à la puissance $\alpha$ (élasticité de la production), mesurée de manière approchée par la part du travail dans la production du secteur dans la région, selon l'équation suivante:

$$\left(\frac{Y_{ij} - \Delta Y_{ij}}{Y_{ij}}\right) = \left(1 - \frac{\Delta L_{ij}}{L_{ij}}\right)^{\alpha_{ij}}. \quad (1)$$

La part du travail est obtenue par approximation en divisant la rémunération totale des travailleurs du secteur (coût du travail majoré des avantages accessoires au salaire et des charges) par la valeur de la production sectorielle, nette des taxes sur la valeur ajoutée. Pour estimer le modèle, nous utilisons des microdonnées issues des enquêtes nationales sur la population active et sur le revenu et les dépenses des ménages en pondérant les échantillons afin de calculer la rémunération totale par secteur. Nous considérons que la diminution de la valeur ajoutée due au COVID-19 est égale à la diminution proportionnelle multipliée par la valeur ajoutée produite par le secteur dans la région en 2019, au prorata du pourcentage de l'année que représente la période.

Dans les comptes nationaux, les chiffres sur la valeur ajoutée sont publiés au niveau à deux chiffres de la PSIC pour les statistiques nationales et au niveau à un chiffre pour les statistiques régionales. Les microdonnées des enquêtes sur la population active et sur le revenu et les dépenses des ménages permettent de calculer, au sein de chaque secteur désigné par un code à un chiffre, la répartition du travail au niveau à deux chiffres par région. Pour obtenir la valeur ajoutée par région au niveau à deux chiffres de la PSIC, nous supposons que, au sein de chaque secteur désigné par un code à un chiffre, la valeur ajoutée suit la répartition du travail dans le secteur désigné par un code à deux chiffres dans chaque région, multipliée par la production nationale moyenne des travailleurs du secteur en question.

## 3.3. Chocs nets

Considérer que les pertes de valeur ajoutée dues au choc d'offre correspondent aux pertes observées à la suite des restrictions imposées aux travailleurs et les ajouter aux pertes dues au choc de demande conduit à surestimer les pertes, puisque la baisse de la demande aurait très bien pu entraîner une diminution de la production dans le secteur, la région et la période même en l'absence de restrictions. À titre d'exemple, en imaginant que les activités touristiques soient interdites et que la demande de services touristiques soit nulle, la diminution de la valeur ajoutée dans ce secteur serait de 100 pour cent et non de 200 pour cent. Pour éviter ce type de double comptage, pour un secteur, une région et une période donnés, nous retenons soit la baisse de valeur ajoutée induite par le choc de demande soit, si elle est plus élevée, celle induite par le choc d'offre. À noter que nous adoptons ainsi une approche prudente du calcul des pertes de valeur ajoutée, étant donné que les effets sur l'offre et sur la demande sont considérés comme des substituts au lieu d'être additionnés. Or, il est possible que les pertes réelles soient cumulatives plutôt que substitutives. Ces variations observées au cours de chaque période et région sont proratisées en fonction de la proportion de l'année que représente la période. La somme de ces variations permet d'obtenir les variations annuelles par région, elles-mêmes additionnées pour obtenir les variations nationales.

## 3.4. Analyse de l'emploi et du revenu

Nous cherchons à déterminer comment le coût des restrictions se répartit entre les groupes de revenu et entre les sexes. À cette fin, nous devons effectuer des simulations des chocs sur le revenu, ce qui est relativement simple pour le revenu salarial – une baisse de la demande de main-d'œuvre correspond à une baisse de revenu en supposant qu'il soit possible de licencier facilement ou de modifier facilement la durée de travail de la plupart des salariés et que la majorité d'entre eux n'aient pas accès à des prestations de chômage. Dans le cas des personnes qui travaillent à leur compte et des chefs d'entreprise, une réduction de la valeur ajoutée correspond à une baisse de revenu. Pour tenir compte de ce type de production, nous calculons la proportion de travailleurs d'un secteur et d'une région qui travaillent pour le compte d'autrui et la multiplions par la diminution du travail, tandis que nous calculons la part de travailleurs d'un secteur et d'une région qui ne travaillent pas pour le compte d'autrui et la multiplions par la diminution de la valeur ajoutée. Nous additionnons ensuite ces valeurs pour obtenir la variation proportionnelle du revenu du côté de l'offre.

Du côté de la demande, une baisse de la demande de production est transformée en une baisse de la demande de main-d'œuvre au moyen d'une fonction de production de Cobb-Douglas, comme suit:

$$\frac{\Delta L_{ij}}{L_{ij}} = \left(1 - \frac{\delta_{ij}}{Y_{ij}}\right)^{\frac{1}{\alpha_{ij}}}. \tag{2}$$

Dans ce cas, la proportion de la production qui se poursuit est élevée à un exposant égal à l'inverse de la part du travail, et le terme $\delta/Y$ représente la réduction proportionnelle de la valeur ajoutée. Nous retenons pour chaque secteur, région et période la réduction proportionnelle du revenu la plus élevée entre celle induite par un choc de demande et celle induite par un choc d'offre. Ensuite, la réduction obtenue pour chaque période est proratisée en fonction du pourcentage de l'année que représente la période. Les résultats sont additionnés et permettent d'obtenir la réduction proportionnelle annuelle.

Pour transformer les variations proportionnelles en variations absolues de l'emploi, nous les multiplions par le nombre d'hommes et de femmes travaillant dans chaque secteur et région puis additionnons les résultats.

Pour le revenu, nous multiplions les variations proportionnelles par le revenu pondéré des ménages déclaré pour chaque secteur dans la région dans la vague 2015 de l'enquête sur le revenu et les dépenses des ménages. Ce revenu est retiré à chaque travailleur de manière uniforme pour tous les travailleurs du secteur et de la région, parce qu'il n'est pas possible de déterminer quels sont ceux, dans un secteur et une région, qui risquent le plus de perdre leur emploi. Nous répétons cet exercice pour le revenu des hommes et le revenu des femmes dans chaque secteur et région, ce qui nous permet de mesurer les effets différenciés en fonction du sexe des restrictions imposées à un secteur dans une région sur la base de la part de l'emploi et du revenu par sexe dans chaque secteur et région.

Pour calculer les effets des restrictions sur la pauvreté, à partir des microdonnées issues de la vague 2015 de l'enquête sur le revenu et les dépenses des ménages, nous additionnons le revenu corrigé au niveau des ménages et nous effectuons une pondération en fonction des populations provinciale et nationale. Ensuite, nous calculons le nombre de ménages pauvres à partir du seuil de pauvreté provincial en vigueur en 2015 et d'autres indicateurs de pauvreté et nous le comparons aux valeurs réelles de 2015 en faisant divers regroupements afin d'examiner les effets des restrictions sur différents sous-groupes d'intérêt.

## 4. Résultats

D'après les résultats de l'estimation de l'équation (1), l'activité économique a sensiblement diminué, de 13,5 pour cent à l'échelle nationale (tableau 1). Cette contraction est concentrée dans les régions plus urbaines, qui ont été soumises à des confinements plus longs et plus stricts, et dans les régions dépendantes de secteurs durement touchés, comme le tourisme. À noter que la baisse prédite par la modélisation est conforme au recul effectif de la croissance du PIB, qui a atteint 15,5 points de pourcentage, la croissance étant passée de 6 pour cent

**Tableau 1. Estimation par simulation de la baisse de l'activité économique induite par la pandémie de COVID-19 et les restrictions imposées pour la contenir aux Philippines, par région (en millions de pesos philippins)**

| Région | Niveau de réf. (2019) | Perte de valeur ajoutée en 2020 | Équivalent en pourcentage |
|---|---|---|---|
| Région I (Ilocos) | 650 542 | 95 187 | 14,6 |
| Région II (vallée du Cagayan) | 396 522 | 52 317 | 13,2 |
| Région III (Luzon centrale) | 2 173 084 | 305 165 | 14,0 |
| Région IV-A (CALABARZON) | 2 855 689 | 400 795 | 14,0 |
| MIMAROPA | 376 103 | 54 043 | 14,4 |
| Région V (Bicol) | 556 981 | 78 335 | 14,1 |
| Région VI (Visayas occidentales) | 912 135 | 142 501 | 15,6 |
| Région VII (Visayas centrales) | 1 262 484 | 239 350 | 19,0 |
| Région VIII (Visayas orientales) | 463 608 | 46 392 | 10,0 |
| Région IX (péninsule de Zamboanga) | 395 042 | 42 810 | 10,8 |
| Région X (Mindanao du Nord) | 877 080 | 75 570 | 8,6 |
| Région XI (Davao) | 919 092 | 114 284 | 12,4 |
| Région XII (SOCCSKSARGEN) | 467 502 | 49 821 | 10,7 |
| Région de la capitale nationale (NCR) | 6 235 408 | 816 224 | 13,1 |
| Région administrative de la Cordillère (CAR) | 320 695 | 42 926 | 13,4 |
| Région autonome du Mindanao musulman (ARMM) | 244 840 | 24 960 | 10,2 |
| Région XIII (Caraga) | 304 341 | 32 918 | 10,8 |
| **Total** | **19 411 148** | **2 613 598** | **13,5** |

Note: Les régions sont désignées conformément aux dénominations officielles de l'office national de la statistique philippin (état au 30 juin 2021). Le taux de change utilisé est une moyenne établie à partir des valeurs données par la Banque centrale des Philippines pour 2019-2020 (1 dollar des États.-Unis = 50,71 pesos philippins).

Source: Estimations des auteurs.

en 2019 à –9,5 pour cent en 2020. La contraction issue de la modélisation étant légèrement inférieure à la contraction réelle, il est possible que les résultats obtenus correspondent à des valeurs légèrement sous-estimées.

D’après la simulation, les restrictions ont eu des effets encore plus forts sur la demande de main-d’œuvre et sur l’emploi que sur la valeur ajoutée, et ceux-ci diffèrent sensiblement selon le sexe. Il ressort du modèle que la réduction de la demande de main-d’œuvre est supérieure de 20 pour cent pour les femmes, ce qui s’explique par la surreprésentation de la main-d’œuvre féminine dans certains des secteurs qui ont subi les pertes les plus lourdes, comme celui des agences de voyage et des activités liées à l’emploi (tableau 2). Globalement, l’emploi des femmes a reculé de 27 pour cent et celui des hommes de 22,5 pour cent. On retrouve de tels écarts dans toutes les régions des Philippines.

Les résultats de la simulation montrent également que la baisse de la demande de main-d’œuvre s’est traduite par un recul sensible du revenu des ménages en 2020, le revenu du travail des femmes ayant diminué de 29,1 pour cent et celui des hommes de 26,2 pour cent (tableau 3), ce qui, dans les deux cas, est nettement supérieur à la diminution de la valeur ajoutée nationale modélisée (13,5 pour cent). En conséquence, la réduction relative subie par les femmes est supérieure de plus de 10 pour cent à celle subie par les hommes. Cette tendance nationale se retrouve partout, sauf dans la région de la capitale nationale, où la diminution relative est pratiquement la même pour les femmes et pour les

**Tableau 2. Estimation par simulation de la baisse de la demande de main-d’œuvre induite par la pandémie de COVID-19 et les restrictions imposées pour la contenir aux Philippines**

| Région | Niveau de réf. (2019) | | Baisse de la demande de main-d’œuvre en 2020 | | Équivalent en pourcentage | |
|---|---|---|---|---|---|---|
| | Hommes | Femmes | Hommes | Femmes | Hommes | Femmes |
| Région I (Ilocos) | 1306665 | 778096 | 279273 | 200453 | 21,4 | 25,8 |
| Région II (vallée du Cagayan) | 957307 | 551377 | 155408 | 121503 | 16,2 | 22,0 |
| Région III (Luzon centrale) | 2634589 | 1654403 | 723220 | 518487 | 27,5 | 31,3 |
| Région IV-A (CALABARZON) | 2985655 | 2243622 | 857668 | 717266 | 28,7 | 32,0 |
| MIMAROPA | 806309 | 496966 | 141971 | 118502 | 17,6 | 23,8 |
| Région V (Bicol) | 1473980 | 901111 | 279026 | 230561 | 18,9 | 25,6 |
| Région VI (Visayas occidentales) | 1971874 | 1324941 | 382150 | 330436 | 19,4 | 24,9 |
| Région VII (Visayas centrales) | 1781313 | 1364460 | 492640 | 420389 | 27,7 | 30,8 |
| Région VIII (Visayas orientales) | 1129613 | 683429 | 173071 | 135737 | 15,3 | 19,9 |
| Région IX (péninsule de Zamboanga) | 890688 | 524300 | 140064 | 103945 | 15,7 | 19,8 |
| Région X (Mindanao du Nord) | 1182715 | 744754 | 208621 | 143857 | 17,6 | 19,3 |
| Région XI (Davao) | 1229454 | 716435 | 257032 | 179461 | 20,9 | 25,0 |
| Région XII (SOCCSKSARGEN) | 1076855 | 658210 | 167725 | 132760 | 15,6 | 20,2 |
| Région de la capitale nationale (NCR) | 2709564 | 2223586 | 887634 | 742440 | 32,8 | 33,4 |
| Région administrative de la Cordillère (CAR) | 458424 | 306969 | 80021 | 65878 | 17,5 | 21,5 |
| Région autonome du Mindanao musulman (ARMM) | 889397 | 329524 | 100601 | 49995 | 11,3 | 15,2 |
| Région XIII (Caraga) | 669929 | 395233 | 110759 | 82801 | 16,5 | 21,0 |
| **Total** | **24154330** | **15897418** | **5436882** | **4294471** | **22,5** | **27,0** |

Note: Les régions sont désignées conformément aux dénominations officielles de l’office national de la statistique philippin (état au 30 juin 2021).
Source: Estimations des auteurs.

**Tableau 3. Estimation par simulation de la baisse du revenu des ménages induite par la pandémie de COVID-19 et les restrictions imposées pour la contenir aux Philippines (en millions de pesos philippins)**

| Région | Niveau de réf. (2019) | | Perte de revenu en 2020 | | Équivalent en pourcentage | |
|---|---|---|---|---|---|---|
| | Hommes | Femmes | Hommes | Femmes | Hommes | Femmes |
| Région I (Ilocos) | 105071 | 58869 | 25905 | 15910 | 24,7 | 27,0 |
| Région II (vallée du Cagayan) | 69992 | 38232 | 14297 | 10510 | 20,4 | 27,5 |
| Région III (Luzon centrale) | 284726 | 170700 | 85040 | 55117 | 29,9 | 32,3 |
| Région IV-A (CALABARZON) | 348954 | 235167 | 103655 | 72841 | 29,7 | 31,0 |
| MIMAROPA | 55603 | 33664 | 11345 | 9683 | 20,4 | 28,8 |
| Région V (Bicol) | 104211 | 66988 | 23238 | 19256 | 22,3 | 28,7 |
| Région VI (Visayas occidentales) | 139986 | 100753 | 31413 | 27073 | 22,4 | 26,9 |
| Région VII (Visayas centrales) | 154220 | 109405 | 49135 | 37607 | 31,9 | 34,4 |
| Région VIII (Visayas orientales) | 80575 | 53618 | 14350 | 11725 | 17,8 | 21,9 |
| Région IX (péninsule de Zamboanga) | 63450 | 36863 | 12118 | 9087 | 19,1 | 24,7 |
| Région X (Mindanao du Nord) | 96046 | 56769 | 18847 | 12671 | 19,6 | 22,3 |
| Région XI (Davao) | 108506 | 67385 | 24682 | 17321 | 22,7 | 25,7 |
| Région XII (SOCCSKSARGEN) | 71579 | 47866 | 12894 | 10964 | 18,0 | 22,9 |
| Région de la capitale nationale (NCR) | 447074 | 324022 | 143146 | 101761 | 32,0 | 31,4 |
| Région administrative de la Cordillère (CAR) | 41539 | 28345 | 9189 | 7386 | 22,1 | 26,1 |
| Région autonome du Mindanao musulman (ARMM) | 49254 | 18093 | 6643 | 4153 | 13,5 | 23,0 |
| Région XIII (Caraga) | 54582 | 34587 | 10691 | 8209 | 19,6 | 23,7 |
| **Total** | **2275366** | **1481327** | **596589** | **431274** | **26,2** | **29,1** |

Note: Les régions sont désignées conformément aux dénominations officielles de l'office national de la statistique philippin (état au 30 juin 2021). Le taux de change utilisé est une moyenne établie à partir des valeurs données par la Banque centrale des Philippines pour 2019-2020 (1 dollar des États.-Unis = 50,71 pesos philippins).

Source: Estimations des auteurs.

hommes. En valeur absolue, les pertes subies par les hommes sont supérieures à celles subies par les femmes, ce qui s'explique en grande partie par le fait que la rémunération et le taux de participation des hommes étaient plus élevés avant la pandémie de COVID-19.

Une grande partie de ces pertes de revenu a surtout été subie par les populations pauvres et vulnérables. D'après le modèle, la pauvreté a quasiment doublé aux Philippines par rapport au scénario de référence sans COVID-19, avec une hausse de 73,3 pour cent à l'échelle nationale sous l'effet des restrictions adoptées en 2020 (tableau 4). Cette augmentation de la pauvreté est plus marquée dans les zones urbaines que dans les zones rurales, ce qui constitue une différence avec bon nombre des crises économiques survenues dans le passé. Ce phénomène s'explique en partie par les secteurs touchés par les variations de la demande dues à la pandémie, mais surtout par le fait que les restrictions ont davantage concerné les zones relativement urbanisées. Les ménages ayant une femme à leur tête ont été plus durement touchés que les autres, mais cette disparité est constatée dans les zones rurales plus que dans les zones urbaines. Les chocs de demande ont un impact plus fort sur la pauvreté des ménages ayant une femme à leur tête, même en l'absence de restrictions. Les restrictions ont cependant creusé l'écart.

**Tableau 4. Estimation par simulation de la variation de la pauvreté induite par la pandémie de COVID-19 et les restrictions imposées pour la contenir aux Philippines, par type de ménage**

| Type de ménage | | Taux de pauvreté relative (%) | Inégalités de revenu | |
|---|---|---|---|---|
| | | | Coeff. de Gini | C75/C25 |
| Tous les ménages | Pandémie seulement | 140,4 | 103,5 | 102,5 |
| | Restrictions | 173,3 | 100,1 | 94,6 |
| Ménages urbains | Pandémie seulement | 174,9 | 105,3 | 108,4 |
| | Restrictions | 237,2 | 103,9 | 102,5 |
| Ménages ruraux | Pandémie seulement | 131,2 | 103,1 | 101,0 |
| | Restrictions | 156,2 | 98,3 | 96,0 |
| Ménages ayant une femme à leur tête | Pandémie seulement | 147,4 | 102,0 | 102,6 |
| | Restrictions | 190,4 | 102,1 | 99,9 |
| Ménages ayant un homme à leur tête | Pandémie seulement | 139,3 | 102,5 | 102,0 |
| | Restrictions | 170,7 | 97,9 | 94,3 |
| Ménages urbains ayant une femme à leur tête | Pandémie seulement | 168,7 | 105,2 | 106,6 |
| | Restrictions | 232,0 | 106,9 | 105,4 |
| Ménages urbains ayant un homme à leur tête | Pandémie seulement | 176,2 | 104,6 | 109,8 |
| | Restrictions | 238,3 | 101,8 | 102,5 |
| Ménages ruraux ayant une femme à leur tête | Pandémie seulement | 138,0 | 102,0 | 101,0 |
| | Restrictions | 171,9 | 100,5 | 96,8 |
| Ménages ruraux ayant un homme à leur tête | Pandémie seulement | 130,3 | 103,6 | 101,0 |
| | Restrictions | 154,1 | 97,7 | 95,7 |

Note: C75/C25 désigne le rapport interquartile, établi en rapportant le revenu au 75$^{e}$ centile de la répartition au revenu au 25$^{e}$ centile.

Source: Estimations des auteurs.

## 5. Étude de cas: coûts immédiats pour les parents et les enseignants

Les fermetures d'écoles ont eu des retombées supplémentaires sur le marché du travail, parce qu'elles ont entraîné des pertes d'emploi dans l'enseignement privé et une diminution de revenu pour les parents contraints de s'occuper de leurs enfants. D'après les statistiques du ministère de l'Éducation datant de septembre 2020, le nombre d'inscriptions dans les écoles privées a chuté de 48 pour cent au cours de l'année scolaire 2020/2021 (Luz, 2020). Selon le Conseil de coordination des associations de l'enseignement privé, les écoles privées emploient quelque 409 757 personnes, enseignants et autres personnels confondus (Magsambol, 2020). Il ressort de l'enquête sur la population active que deux tiers des enseignants sont des femmes. Nous nous appuyons sur les données de l'enquête sur la population active et de l'enquête sur le revenu et les dépenses des ménages et postulons que 30 pour cent de ces salariés ont perdu leur emploi, ce qui équivaut à une perte d'environ 123 000 emplois au cours de l'année scolaire 2020/2021 et à une diminution de salaire estimée à 16 milliards de pesos philippins (ci-après «pesos»).

S'agissant des pertes de revenu subies par les parents, nous utilisons les données de l'enquête sur la population active et de l'enquête sur le revenu et les dépenses des ménages pour isoler tous les ménages comptant des enfants de moins de 12 ans et dans lesquels les deux parents travaillent. Nous postulons qu’un adulte doit avoir un niveau scolaire au moins égal au deuxième cycle du secondaire pour pouvoir aider un enfant à suivre sa scolarité en ligne. Nous considérons également que, lorsque les deux parents travaillent et qu’il n’y a pas de grands-parents de moins de 80 ans ayant un niveau scolaire au moins égal au deuxième cycle du secondaire, l’un des deux parents, en l’occurrence celui ayant le revenu d’activité le plus faible, est obligé de rester au foyer. Dans ces ménages, environ 92 pour cent des pères perçoivent un revenu supérieur à celui des mères, lesquelles ont en outre un niveau d’études correspondant à 9,5 années contre 8,5 années pour les pères. Autrement dit, la mère est le parent le plus susceptible d’arrêter de travailler pour encadrer les enfants qui suivent leur scolarité en ligne. Environ un tiers des ménages de l’échantillon remplissent les critères retenus. D’après notre simulation, la perte de revenu des ménages s’établit à 6 pour cent, soit 225 milliards de pesos pour une année scolaire. Cette perte correspond pour l’essentiel (207 milliards de pesos) au salaire perdu par les femmes contraintes de rester au foyer pour s’occuper des enfants et de leur scolarité. En conséquence, le revenu des femmes régresse de 14 pour cent, tandis que celui des hommes diminue de 0,1 pour cent. Par ailleurs, les enseignantes du secteur privé ont perdu 10,7 milliards de pesos de salaire en raison de la fermeture des écoles; et leurs homologues de sexe masculin, 5,3 milliards de pesos.

À moyen ou long terme, une interruption prolongée des cours en présentiel risque de compromettre la capacité des jeunes Philippins à devenir des travailleurs productifs. La menace est plus grande pour les ménages les plus pauvres, qui sont ceux qui éprouvent le plus de difficultés à aider leurs enfants à suivre leur scolarité à distance. Le pourcentage d’enfants qui ont cessé d’aller à l’école a été plus élevé aux Philippines que dans les autres pays de la région, et le pourcentage de ceux qui ont pu suivre des cours en ligne (lorsqu’il en existait) est resté relativement faible (21 pour cent) en raison de la mauvaise qualité de la connexion à Internet et de l’absence d’équipement informatique (Morgan et Trinh, 2021). Étant donné que durant la crise financière asiatique de 1998-1999, aux Philippines, le taux de décrochage scolaire a été plus élevé parmi les filles que parmi les garçons, il est possible que les filles ne retournent pas à l’école aussi vite que les garçons lorsque les écoles fermées à cause du COVID-19 rouvriront (Banque mondiale, 2020). Le fait que les pertes d’emplois soient plus nombreuses parmi les femmes et le décrochage scolaire plus fréquent parmi les filles, couplé avec l’augmentation des grossesses non désirées dont les médias se font l’écho, risque non seulement d’anéantir les progrès accomplis en matière d’égalité, mais aussi d’affaiblir la reprise économique en réduisant les chances d’une partie importante de la population d’accéder à un emploi qualifié.

## 6. Conclusion et enseignements pour l’action publique

Aux Philippines, la pandémie de COVID-19 et les mesures adoptées pour l’endiguer ont fortement nui à la capacité des travailleurs, à commencer par celle des femmes, à exercer une activité et à apporter leur contribution à l’économie. La pandémie a mis en évidence la vulnérabilité des plus pauvres, les femmes parmi eux notamment, face à la crise économique. Nos résultats montrent en effet qu'aux Philippines les personnes à faible revenu ont supporté une part plus importante des conséquences de la crise. La pandémie révèle au grand jour les failles du système existant (normes sociales, services, infrastructures) ainsi que l'écart dans les conditions d'existence effectives selon le sexe et le groupe de revenu. D'après notre analyse, la pauvreté va connaître à court terme une forte augmentation, qu'il faudra des années pour effacer. Cette hausse de la pauvreté aura elle-même une incidence sur les stratégies mises en œuvre pour stimuler la reprise.

À la date de rédaction de l'article, il était prévu que les mesures de confinement à l'origine d'une grande partie de ces phénomènes perdurent en 2021. Toutefois, les conséquences décrites ici n'ont rien d'une fatalité. D'autres solutions qu'un confinement total et la fermeture généralisée des écoles pourraient être envisagées pour lutter contre la pandémie sans sacrifier l'avenir des femmes et des pauvres. Chen et ses coauteurs (2020), par exemple, montrent que des mesures de distanciation physique ciblées, le traçage des cas contacts et les congés-maladie indemnisés contribuent plus efficacement à la lutte contre la pandémie que les fermetures d'écoles et la restriction des mouvements. Faire davantage appel à ce type de stratégies pourrait réduire l'impact de la pandémie sur les publics défavorisés.

Étant donné les conséquences négatives directes et indirectes de la pandémie et des mesures de confinement sur l'égalité entre hommes et femmes, il faudrait que les stratégies de relance tiennent compte des liens entre les normes sociales traditionnelles et les inégalités de genre afin que la reprise soit plus égalitaire. Bien que les Philippines aient accompli de grandes avancées sur le plan de l'égalité entre hommes et femmes ces dernières décennies, la conception traditionnelle du rôle des femmes en tant que procréatrices est pour beaucoup dans la faiblesse du taux d'activité féminine et dans le risque de pauvreté auquel sont exposées les femmes dans le contexte de la pandémie. Les pouvoirs publics philippins ont fait preuve de volontarisme lorsqu'il s'est agi de garantir une couverture d'assurance-maladie pour le COVID-19 ou d'instituer des prestations en espèces et des aides alimentaires en faveur des ménages pauvres (Park et Inocencio, 2020). Le COVID-19 risquant de réduire à néant les progrès accomplis en matière d'égalité entre hommes et femmes, il faut que les autorités cherchent encore davantage, dans leurs stratégies de relance, à favoriser une reprise axée sur le soin, alliant une protection sociale généreuse et des mesures contracycliques visant à stimuler la demande totale et des investissements dans les services de soins essentiels. L'investissement public dans les services d'accueil des enfants et de prise en charge des personnes dépendantes est de nature à créer des millions d'emploi, à la fois directement dans ces secteurs, mais aussi indirectement en favorisant l'activité économique dans certains des pans de l'économie les plus durement touchés, comme la distribution, les soins de santé et la restauration, dans lesquels les femmes sont majoritaires. Il faudra atténuer les effets des mesures de confinement déjà adoptées, notamment par un soutien global susceptible d'aider les femmes à accéder à un travail décent et à des possibilités de formation – des services de garde d'enfants abordables et de bonne qualité sont des exemples de ce soutien. Il est aussi indispensable de prendre des mesures pour que les filles, plus exposées au risque de décrochage scolaire, soient rescolarisées.

Il faut par ailleurs impérativement que les mesures prises pour lutter contre la pandémie et favoriser la reprise permettent aux femmes et aux filles d'accéder à des services de santé sexuelle et procréative afin de réduire le nombre, actuellement élevé, de grossesses non désirées et de mariages précoces et de protéger la santé maternelle et infantile. Outre leurs répercussions négatives sur la santé des femmes et des enfants, les grossesses non désirées et les grossesses parmi les adolescentes accentuent l'exposition des femmes au risque de pauvreté et réduisent leur chance d'accéder au marché du travail ou de faire des études. Consciente que la pandémie ne doit pas empêcher d'agir contre les grossesses des adolescentes, la commission de la population des Philippines a lancé de nouvelles initiatives visant à sensibiliser et à mobiliser les services de l'administration locale, les prestataires de soins et les populations. Il est indispensable que ce type d'initiatives monte en puissance. La sanctuarisation des budgets publics, le développement des services de télémédecine et la recherche de moyens supplémentaires pour améliorer l'accès aux services de planification familiale pendant les confinements sont autant de pistes qui permettront de protéger la santé et le bien-être des femmes et de leurs enfants et, en conséquence, de contrecarrer les répercussions négatives des confinements sur l'emploi et le salaire des femmes.
